# Prevalence and Associated Factors of Malocclusion among Preschool Children in Huizhou, China: A Cross-Sectional Study

**DOI:** 10.3390/healthcare11071050

**Published:** 2023-04-06

**Authors:** Lude Lin, Wanting Chen, Dejun Zhong, Xiayuan Cai, Jieyi Chen, Fang Huang

**Affiliations:** 1 Hospital of Stomatology, Sun Yat-sen University, Guangzhou 510055, China; 2 Guangdong Provincial Key Laboratory of Stomatology, Guangzhou 510080, China; 3 Guanghua School of Stomatology, Sun Yat-sen University, Guangzhou 510080, China; 4 Huizhou Second Maternal and Child Healthcare Hospital, Guangdong Medical University, Huizhou 516001, China

**Keywords:** malocclusion, occlusal traits, dental care, primary dentition, child

## Abstract

This survey examined the prevalence of malocclusion and its associated factors in 3- to 5-year-old children in Huizhou, China. Children were recruited from 21 kindergartens using a multistage sampling method. Two examiners performed oral examinations on the children, assessing occlusal traits, including primary molar and canine relationship, overjet, crossbite, overbite, anterior open bite, scissor bite, midline deviation, crowding, and spacing. Caries experience was recorded. Self-administered questionnaires were distributed to collect background information and oral health-related habits. Chi-square test, Mann–Whitney U test, and binary logistic regression were conducted. The study invited 1485 children and eventually recruited 1454 (52.9% boys; response rate: 97.9%). The prevalence of malocclusion was 68.3%, and deep overbite was found in 48.6% of the children. Children who had lip-biting habits had a higher prevalence of deep overbite than those who did not (OR = 2.36, *p* < 0.05). Children who had digit-sucking habits and dental caries in anterior maxillary teeth had a lower prevalence of deep overbite than those who did not (OR = 0.73 and 0.70, respectively, *p* < 0.05). In conclusion, over half of the children in Huizhou who participated in the study had malocclusion. Dental caries in anterior maxillary teeth, digit-sucking, and lip-biting were the associated factors of deep overbite.

## 1. Introduction

Malocclusion is a developmental disorder characterized by abnormal relationships between teeth alignment and dental arches. It is considered the third most prevalent dental public health problem and has an influence on oral health-related quality of life [1]. Severe malocclusion may cause both physiological and psychological conditions, such as deficiencies in masticatory function, speech articulation, and aesthetic appearance, thereby exerting a negative impact on self-esteem and social interactions in the long term [2]. The etiology of malocclusion is multifactorial, and the condition can result from genetic and environmental influences. The skeletal pattern, determined by genes, is regarded as the most important determinant of malocclusion [3]. Environmental factors, such as oral habits (including non-nutritive sucking habits, tongue-thrusting, and mouth-breathing), dental caries, and changes in feeding practices may contribute to the increased prevalence of malocclusion [4].

It has been found that the prevalence of malocclusion in primary dentition varies greatly across different countries. In Brazil, the prevalence of malocclusion in primary dentition was 63.2% in 2010, as reported by the Brazil National Oral Health Survey [5]. In a Chinese national survey conducted in 2000, the prevalence of malocclusion among 5-year-old Chinese preschool children was reported to be 51.84% [6]. In 2017, Sepp found that only 7.2% of preschool Estonians had a symmetrical flush terminal plane and Class I in deciduous canines, an overjet of 1–3 mm, an overbite of 1–3 mm, no crowding, scissor bite, or crossbite [7]. In southwest Germany, over 58% of 5-year-old children were found to exhibit malocclusion traits [8].

Over the last few decades, there has been a growing interest in the role of primary dentition in determining the alignment of permanent teeth. However, most epidemiological studies have only focused on the occlusal traits of permanent dentition. Some cohort studies have suggested that malocclusion of primary dentition is a determinant of malocclusion of permanent dentition [9,10]. Peres found that children with open bite and canine malocclusion during their primary dentition are more likely to require orthodontic treatment of their permanent dentition [11]. Although this does not occur in all cases due to individual differences, severe malocclusion in primary dentition can have negative effects on permanent dentition. Therefore, it is necessary to understand the prevalence of malocclusion conditions and their associated factors during the development of primary dentition in order to implement preventive strategies.

Huizhou is a second-tier coastal city in the fast-growing Pearl River Delta of Guangdong Province in southeastern China, with a population of approximately 6 million [12]. Around 80% of Huizhou residents are Hakka people [13]. The gross domestic product was 70,191 Chinese yuen (~USD 9862) per capita in Huizhou city in 2021, an amount lower than the national average (~USD 12,551) [12,14]. Currently, there is a lack of information about malocclusion in children in Huizhou. The aim of the present study was to investigate the prevalence of malocclusion and its associated factors in primary dentition among 3- to 5-year-old children in Huizhou, China. 

## 2. Materials and Methods

This cross-sectional study was conducted between March and May 2022. Ethical approval was obtained from the Medical Ethics Committee of the Hospital of Stomatology, Sun Yat-sen University (KQEC-2021-39-03), and written consent was obtained from the parents of the participating children. This study was conducted in accordance with the relevant guidelines and regulations of the Declaration of Helsinki of 2013 and reported following the Statement of Strengthening the Reporting of Observational Studies in Epidemiology (Supplementary File S1) [15].

### 2.1. Sample Size Calculation and Sample Selection 

The sample size was calculated according to the following formula: *n* = Z^2^ × P × (1 – P)/d^2^. The Z statistic was based on the confidence level, P was the prevalence rate, and d was the precision [16]. The estimated prevalence of malocclusion was 45.5% [17]. The confidence level was set at 95%, and the confidence interval (CI) was set at 10% (40.5 to 50.5%). The sample size was computed to be 382. Most kindergartens in China have a 3-year program (K1, K2, and K3). Assuming a response rate of 80%, this study recruited 478 children from each year and 1434 in total. 

This study adopted the multistage sampling method. Huicheng District has eight main sub-districts, namely Heinanan, Jiangbei, Jiangnan, Longfeng, Qiaodong, Qiaoxi, Shuikou, and Xiaojinkou. These were regarded as clusters. All the districts had public and private kindergartens. The ratio of children invited from public and private kindergartens in each cluster was determined by the ratio of public to private kindergartens in each cluster and the ratio of the population distribution in the eight clusters. In the first stage, private and public kindergartens in each cluster were stratified. Then, they were numbered sequentially and selected using a simple random sampling method. In the second stage, one class from each year (K1, K2, and K3) was chosen from the selected kindergarten using a simple random sampling method. In the third stage, all the children in the selected class were invited using a cluster sampling method. Children from kindergartens were invited until the desired number of participants in the cluster was achieved.

### 2.2. Inclusion and Exclusion Criteria

All 3- to 5-year-old children in the selected classes were invited to participate in the study. Children with (1) permanent teeth, (2) orthodontic treatment history, (3) craniofacial anomalies, (4) serious systemic diseases, and (5) difficulty in cooperating were excluded from this study.

### 2.3. Questionnaire Survey

A close-ended questionnaire was developed based on previous studies and distributed to the parents of the participating children [18,19,20,21]. It consisted of 20 questions and included the following three parts:

- Part I: The child’s background information, age, and sex;
- Part II: The child’s deleterious oral habits: digit-sucking, lip-biting, tongue-thrusting, object-biting, bruxism, unilateral chewing, and mouth-breathing;
- Part III: The child’s feeding pattern: infant feeding practice in the first 6 months of life, duration of breastfeeding and bottle feeding, pacifier use, delivery method, and gestational age at delivery.

Parents completed the questionnaires without any assistance.

### 2.4. Oral Examination

Two examiners (LL and WC) were trained by an experienced pediatric dentist (FH) before the study. These examiners and two data recorders first attended a training workshop in November 2021, organized by FH and JC, and received the research proposal and written instructions for the clinical examination. The next day after the workshop, the researchers and data recorders went to a kindergarten for further training and calibration. During the oral examination, a graded periodontal probe, a Community Periodontal Index probe, and a portable lightning system were used. The single gold standard examiner (FH) conducted a demonstration examination on one child, and each examiner then examined five children for practice. After training, 20 preschool children who fulfilled the inclusion and exclusion criteria were selected for calibration. Each subject was examined for around 5 min by three researchers, and two examiners (LL and WC) examined all subjects again at 30-min intervals. The classification of different types of occlusal traits and the diagnosis of dental caries were recorded to calculate the inter- and intra-examiner agreement. A minimal level of inter- and intra-examiner reliability was set at a kappa value of 0.75. Both examiners maintained excellent inter- and intra- examiner agreement, with kappa values above 0.9 for each type of occlusal trait and dental caries.

After the study began, the trained examiners performed oral examinations in kindergartens with the assistance of two trained data recorders using the same probes and lightning system. Occlusal traits were assessed in participants whose teeth were in occlusion. The diagnostic criteria were as follows [22]:

#### 2.4.1. The Sagittal Relationship of the Second Primary Molars

- Flush terminal plane: The distal surfaces of the maxillary and mandibular second primary molars were in the same vertical plane.
- Distal step: The distal surface of the mandibular second primary molar lay distal to that of the maxillary second primary molar.
- Mesial step: The distal surface of the mandibular second primary molar lay mesial to that of the maxillary second primary molar.
- The sagittal relationship of the primary molars was recorded as distal or mesial step if the flush terminal plane was on one side and the distal or mesial step was on the other side.

#### 2.4.2. The Sagittal Relationship of the Primary Canines

- Class I: The distal surface of the mandibular primary canine and cusp tip of the maxillary primary canine were on the same vertical plane.
- Class II: The distal surface of the mandibular primary canine lay distal to the cusp tip of the maxillary primary canine.
- Class III: The distal surface of the mandibular primary canine lay mesial to the cusp tip of the maxillary primary canine.
- The sagittal relationship of the primary canines was recorded as class II or III if class I was on one side and class II or III was on the other side.

#### 2.4.3. Sagittal Anomalies

- Overjet (mm): Examiners measured the distance between the incisal edge of the most protruded upper primary incisor and labial surface of the corresponding lower primary incisor, parallel to the occlusal plane. Overjet was categorized as normal (>0 mm and ≤4 mm), increased (>4 mm), and edge-to-edge (upper and lower incisal edges met edge-to-edge) incisor relationship.
- Anterior crossbite was diagnosed when any upper primary incisor or canine was positioned inside the lingual surfaces of the lower front teeth.

#### 2.4.4. Vertical Anomalies

- Deep overbite was diagnosed when the coverage of the lower incisors by the most protruded upper primary incisor was more than half.
- Anterior open bite was diagnosed if no vertical overlap was found between the upper and lower primary incisors when the posterior teeth were in contact.

#### 2.4.5. Transversal Anomalies

- Posterior crossbite was diagnosed when any lower primary posterior tooth was placed buccal to the upper primary molars. Both unilateral and bilateral posterior crossbites were recorded.
- Scissor bite was diagnosed if one or more maxillary molars were occluded buccally to the buccal surfaces of the mandibular molars.
- Midline deviation was diagnosed if the midline of the mandibular primary incisors had a deviation ≥2 mm from that of the maxillary primary incisors.

#### 2.4.6. Space Discrepancies

- Crowding was diagnosed if overlapping of erupted primary teeth was >2 mm.
- Spacing was diagnosed if the generalized space between the primary teeth was >2 mm.

Dental caries were diagnosed using the criteria recommended by the World Health Organization (WHO) [23]. Decayed, missing, and filled teeth were designated as having caries. Duplicate examination of 5% of the samples was carried out for occlusal traits and dental caries during oral examination on the same day to assess inter- and intra-examiner agreement. An excellent inter- and intra-examiner agreement was maintained.

Children with one or more of the following oral conditions were considered to have malocclusion: increased overjet, edge-to-edge incisor relationship, anterior crossbite, deep overbite, anterior open bite, posterior crossbite, scissor bite, midline deviation, and crowding. 

### 2.5. Statistical Analysis

The examiners entered the data into a personal computer using Microsoft Office Excel 2013, and two field assistants went through the data again to avoid mis-inputs. Some data were missing for privacy reasons and were thus replaced by the median. Statistical Package for Social Science version 26.0 (SPSS Inc., Chicago, IL, USA) was used for data analysis. Inter-examiner and intra-examiner reproducibility were assessed using intra-class correlation coefficient. Data were summarized as frequencies and percentages. The intra-class correlation coefficients for outcomes were calculated to be 0.03 at class level (*p* = 0.071), 0.02 at kindergarten level (*p* = 0.184), and less than 0.01 at sub-district level (*p* = 0.473). The clustering effect can be ignored. Chi-square test was used to assess the statistical significance of the differences between the studied variables and malocclusion. The Mann-Whitney U test was used to assess the differences between the distribution of dmft score and malocclusion because the dmft score was not normally distributed. All factors with a *p* value of less than 0.1 in the Chi-square test were included in the binary logistic regression analysis. Malocclusion was considered the dependent variable, and odds ratios (OR) along with 95% CIs were calculated. The level of statistical significance for all tests was set at *p* < 0.05.

## 3. Results

This study enrolled 1485 children from 21 kindergartens. Among them, 21 children had no parental consent form and 10 were uncooperative or absent during the examination. Therefore, the study eventually recruited 1454 preschool children, with a response rate of 97.9% (1454/1485). Of these, the mean age of the participants was 4.3 (standard deviation (SD): 0.7) years, and 769 children (52.9%) were boys. 

Only 461 (31.7%) children had normal occlusion, while 993 (68.3%) children had one or more types of malocclusion. A decreasing trend in the rate of malocclusion with age was observed, from 78.2% at age 3 to 63.2% at age 5 (*p* = 0.001, Table 1). Sex was not significantly associated with the prevalence of malocclusion (*p* = 0.300, Table 1).

The prevalence of occlusal traits among the Huizhou preschool children is shown in Table 2. Regarding primary molar relationships, more than half of the children had flush terminal plane (58.3%), and around two-thirds had class I canine relationships (63.8%). 

Deep overbite (48.6%) in the vertical direction was the malocclusion trait with the highest prevalence. Increased overjet (12.5%) and crowding (10.5%) were the second and third most common types of malocclusion. Posterior crossbite and scissor bite were not common (0.1%). Approximately half (53.0%) of the children had spacing. A decreasing trend with age was observed for the prevalence rates of canine class III and anterior crossbite (*p* < 0.05, Table 2).

### 3.1. Association between Dental Caries and Malocclusion

Dental caries were found in 1068 children (73.5%). The prevalence of dental caries was 57.1%, 71.7%, and 81.0% among 3-, 4-, and 5-year-old children, respectively. The overall mean (SD) dmft score was calculated to be 5.15 (5.14), with a mean (SD) dt score of 5.01 (5.04), a mean (SD) mt score of 0.01 (0.09), and a mean (SD) ft score of 0.14 (0.69). The mean (SD) dmft score was 3.32 (4.28), 4.91 (5.07), and 6.05 (5.31) among 3-, 4-, and 5-year-old children, respectively. The prevalence of dental caries in different tooth locations was 38.9% for mandible molars, 33.3% for maxillary anterior teeth, 27.9% for maxillary molars, and 6.6% for mandible anterior teeth. Over half (55.9%) of the maxillary central incisors had dental caries. The prevalence of children with missing teeth was 0.8%, and nearly all (91.7%) of the missing teeth were maxillary central incisors. The prevalence of dental caries was related to malocclusion (*p* < 0.001). The prevalence of malocclusion among children with dental caries was 65.4%, and among children without dental caries was 76.4%. Three- and four-year-old children with dental caries had a lower prevalence of malocclusion (*p* = 0.012 and *p* = 0.026, respectively). The prevalence of malocclusion among 3-year-old children with dental caries was 71.1%, and without dental caries was 87.7%. The prevalence of malocclusion among 4-year-old children with dental caries was 67.4%, and without dental caries was 75.7%. The mean dmft score in children with and without malocclusion was 4.71 and 6.11, respectively. Children with malocclusion had a lower mean dmft score (*p* < 0.001).

Each type of malocclusion was analyzed, and it was found that deep overbite and spacing had statistically significant associations with the prevalence of dental caries (*p* < 0.05, Table 3), while other occlusal traits were not related to dental caries (*p* > 0.05, Table 3). Further analysis found that children with dental caries in anterior maxillary teeth had a lower prevalence of deep overbite (44.9% vs. 54.3%, *p* < 0.001). Additionally, children with spacing had a lower prevalence of dental caries in both anterior and posterior teeth (47.6% vs. 52.4%, *p* < 0.001 and 41.6% vs. 58.4%, *p* < 0.001, respectively). The prevalence of dental caries in anterior teeth among children with spacing was 47.6%, and without spacing was 52.4%. The prevalence of dental caries in posterior teeth among children with spacing was 41.6%, and without spacing was 58.4%. Dental caries in different tooth locations were not significantly related to anterior open bite (*p* > 0.05). Children with dental caries in anterior teeth had no significant relationship with anterior crossbite (*p* = 0.329). Missing teeth had no significant relationship with anterior open bite (*p* = 0.999), anterior crossbite (*p* = 0.999), and deep overbite (*p* = 0.295).

### 3.2. Association between Deleterious Oral Habits and Malocclusion

There was a high prevalence of deleterious oral habits (56.1%) among preschool children in Huizhou. Digit-sucking (28.1%) and bruxism (21.2%) were the most common habits observed, while fewer children engaged in object-biting (13.6%), lip-biting (7.7%), mouth-breathing (7.7%), tongue-thrusting (4.8%), and unilateral chewing (4.1%).

For different types of malocclusion, digit-sucking and lip-biting were found to be statistically related to deep overbite (*p* = 0.021 and *p* < 0.001, respectively, Table 4). Children who engaged in digit-sucking had a lower prevalence of deep overbite when compared to those who did not engage in this habit (43.8% vs. 50.5%, Table 4). Children who engaged in lip-biting had a higher prevalence of deep overbite when compared to those without this habit (67.0% vs. 47.1%, Table 4). Additionally, lip-biting was correlated with a higher prevalence of increased overjet (20.5% vs. 11.8%, *p* = 0.009, Table 4) and crowding (17.0% vs. 9.9%, *p* = 0.021, Table 4). Digit-sucking was associated with a higher prevalence of anterior open bite (2.2% vs. 0.8%, *p* = 0.029).

### 3.3. Association between Feeding/Delivery Methods and Malocclusion

As for the feeding practice in the first 6 months, nearly half of the children (50.9%) were exclusively breastfed, 13.1% were exclusively bottle-fed, and 36.0% were mixed-fed. Children who were breastfed for 6.1–12 months accounted for the largest proportion (40.1%). Most children were bottle-fed for over 18 months (45.3%). The majority of the children (84.1%) had never used a pacifier. Further, 61.9% of children were born via natural labor, and 85.9% were term births. No significant association was found between feeding/delivery methods and deep overbite, increased overjet, crowding (*p* > 0.05, Table 5), and anterior open bite (*p* > 0.05).

### 3.4. Binary Logistic Regression Analysis of Relative Factors and Deep Overbite

The results showed that dental caries in anterior maxillary teeth, digit-sucking, and lip-biting were significantly related to the most prevalent type of malocclusion, deep overbite. Children who had dental caries in anterior maxillary teeth and those who engaged in digit-sucking had a lower prevalence of deep overbite compared to those who did not (OR = 0.70 and 0.73, respectively, Table 6). Conversely, children with a lip-biting habit had a higher prevalence of deep overbite compared to those who did not have the habit (OR = 2.36, Table 6).

## 4. Discussion

This cross-sectional study observed a high prevalence of malocclusion amongst Huizhou preschool children, which was similar to the rate reported in Xi’an, China, in 2016 (66.31%) but higher than the pooled national prevalence for malocclusion in the primary dentition (45.50%) reported in 2018 [17,19]. When compared to peer groups in other countries, the prevalence rate of malocclusion among Huizhou preschool children was close to that of German preschoolers (61.5%) in 2010 but considerably higher than that among Brazilian children reported in 2014 and 2015 (28.4% and 32.5%, respectively) [8,24,25]. However, caution should be exercised when comparing prevalence rates of malocclusion in different countries or cities. Indeed, studies conducted in different countries or cities may adopt different diagnostic criteria, as there is no established and globally recognized definition of malocclusion for primary dentition. For instance, the cutoff point for deep overbite could be two-thirds or half covered [18,19]. In line with other similar publications, molar and canine relationships were not classified as malocclusion in this study [18,26]. The present study provides insight into the occlusal traits among preschool children in Huizhou, China, and is the first study to examine the occlusal condition of preschool children in Hakka settlements. Hakka is a subgroup of the Han Chinese, and there are approximately 80 million Hakka people worldwide today [27]. This population speaks the Hakka dialect, which belongs to the Sino-Tibetan family, and prefers Hakka cuisine, which can be characterized as salty, fragrant, and umami and tends to be hearty and heavy [28,29]. Meizhou, Heyuan, Shaoguan, Qingyuan, Hong Kong, and Macau are large Hakka settlements with a population of over 50 million [30]. Hakka people are also scattered in Indonesia, Malaysia, Thailand, and other overseas countries [27]. So far, no study about malocclusion has been reported in Hakka settlements such as Meizhou and Heyuan. Therefore, the results of this study may help researchers and stakeholders to speculate the probable occlusion conditions among preschool children in these Hakka settlements. However, it is important to note that the study did not include being of Hakka origin as a variable in data analysis in order to avoid recall bias. Some parents were unable to clarify whether they were of Hakka origin or of mixed ethnicity because the Hakka people have a long migration history and have mixed with non-Hakka people for several generations.

This study found that age was related to malocclusion, with a decreasing trend in the rate of malocclusion among 3- to 5-year-old children. Further statistical analysis revealed that the prevalence rate of primary anterior crossbite, as well as class III canine relationship, also decreased significantly with age, affecting the rate of malocclusion. This observation could be partly explained by the self-correction of the primary anterior crossbite and class III canine relationship. A four-year longitudinal study conducted in Japan suggested that primary anterior crossbite could be self-corrected due to the favorable growth of the maxilla in relation to the mandible [31]. Similarly, a study conducted in India reported a decreasing trend in class III canine relationship with increasing age among preschool children [32]. More evidence may be needed to confirm the necessity of interceptive treatment for primary anterior crossbite and class III canine relationship at an early age.

The present study found that deep overbite was the most common type of malocclusion in primary dentition, which is consistent with the results reported in Xi’an in 2017 and Shanghai in 2016 [18,19]. Three factors were associated with a deep overbite. One of these factors was dental caries in deciduous maxillary anterior teeth. This may be partly due to the high prevalence of dental caries in maxillary central incisors and the diagnostic criteria adopted in this study. Primary front teeth are susceptible to dental caries, and the crown could be shortened if the incisal edge is affected [33]. Therefore, children with decayed front teeth might have had a lower chance of being diagnosed with deep overbite in this study. Apart from deep overbite, other studies also reported that dental caries were related to spacing. Cho reported that children without primate space had a higher dmfs score [34]. A cross-sectional study also observed that children without interdental spacing in their primary dentition were at a higher risk for dental caries [35]. The absence of spacing may make it difficult for young children and their parents to practice oral hygiene in contact areas. Therefore, the use of dental floss should be considered for children without general interdental spacing for the purpose of dental caries prevention.

The other two associated factors of deep overbite were lip-biting and digit-sucking. Children with deleterious oral habits have been reported to have a higher incidence rate of malocclusion when compared to those without such habits [4,36]. Lip-biting has been shown to lead to deep overbite and increased overjet because the upper incisors can tip labially and the lower incisors can collapse lingually when the lower lip is wedged between the upper and lower incisors [37,38]. Lip-biting was also correlated with crowding in the present study. However, a study conducted in Nepal focusing on oral habits and malocclusion among 3- to 7-year-olds found no association between crowding and lip habits [39]. The inconsistent finding may be explained by the teeth movement caused by the force of perilabial muscles. Appropriate interventions such as lip training and functional appliances should be considered for children with lip-biting habits [38]. Regarding digit-sucking, it is well recognized that this habit results in greater maxillary arch depth, narrower maxillary arch widths, and a greater prevalence of increased overjet and anterior open bite [20,40]. Therefore, children with this habit may be less likely to be diagnosed with deep overbite. This study’s finding was consistent with that reported by Ling among 2- to 5-year-old Hong Kong children, where digit sucking for more than one year was shown to lead to a higher risk of anterior open bite [41]. Behavior intervention and psychological support should be considered to stop the digit-sucking habit [42].

This study also found that crowding and spacing had a high prevalence, which was consistent with a cross-sectional study conducted by Zhou in Shanghai in 2017 [18]. Crowding is less likely to be self-corrected without any treatment or intervention. The crowding problem may be even worse in permanent dentition as the length of the dental arch decreases during the transition from mixed to permanent dentition [43]. Researchers have suggested that crowding might occur in permanent dentition if there is no space in primary dentition [44]. As leeway and primate spaces are ordinary in primary dentition, it is proposed that spacing suggests appropriate alignment of permanent dentition, and early intervention of spacing is not necessary [17]. Therefore, spacing was not identified as a type of malocclusion in the current study. 

The prevalence rate of increased overjet in this study was lower than that reported in Xi’an (34.9%) in 2017 and Shanghai (33.9%) in 2016 [18,19]. These observations may be attributed to different diagnostic criteria used in those studies, which defined increased overjet as >3 mm in comparison to the 4 mm used in the current study. However, there were no established diagnostic criteria for increased overjet, and the cutoff point (>4 mm) was also used in other studies [20,39].

The open bite was found in only 1.2% of the children in this study, and this rate is comparable to that reported in other studies [18,26]. In the present sample, only 0.1% of the children had posterior crossbite, which was similar to the rates reported in Shanghai (0.3%) in 2016 and India (0.4%) in 2012 [18,45]. However, the result is much lower than the rates reported by Americans and Europeans, which ranged from 7.2% to 20.81% in 2007 [46,47]. A systematic review by Lombardo showed a high prevalence of posterior crossbite in America and Europe, but it could not demonstrate that Caucasian populations exhibited a significantly higher rate than African and Asian populations in primary dentition [1]. Another systematic review by Alhammadi mentioned that Europe showed the highest prevalence of posterior crossbite in permanent dentition [48]. More studies are required to identify the patterns and characteristics of deciduous-dentition malocclusion prevalence by different regions and races.

This study adopted several strategies to maintain the representativeness, reliability, and validity of its results by following the WHO recommendations. This study successfully recruited representative samples, as well as achieved a large enough sample size and a high response rate by multistage sampling. The multistage sampling method saves time, effort, and money and is easy to organize and implement. Two examiners completed sufficient calibration training before the commencement of this study to maintain the reliability and validity of the results, and they obtained good inter- and intra-examiner agreements. Research assistants were also trained with the examiners. To avoid misunderstandings, the researcher’s phone number was printed on the consent form and distributed along with the parental questionnaire, so parents could call the researcher if they had any queries. If missing information was found on the questionnaire, the researcher made phone calls to parents to avoid missing data bias. 

Limitations of the present study must be recognized. One limitation of the current study was that self-administered questionnaires were adopted to determine the presence or absence of deleterious oral habits, as the crowded environment of the schools made it difficult for examiners to detect deleterious oral habits by observation in a relatively short time. Although other studies detected oral habits by self-reported questionnaires, the validity of such data may be influenced by recall bias or misunderstanding [18,19]. Further studies could be conducted to investigate the level of disagreement between the self-reported prevalence of oral habits based on questionnaires completed by parents and the prevalence detected by clinical observation. Another limitation was that this study adopted diagnostic criteria for dental caries according to the WHO recommendations and similar studies; however, it should be noted that the dmft score may be only indicative of the occurrence of dental caries and fail to investigate the severity of the involved tooth decay, such as incipient, moderate, advanced, and severe dental caries [49,50]. This could affect the diagnosis of deep overbite. Criteria that detect and assess the severity of carious teeth should be considered in future studies that aim to investigate the relationship between dental caries and occlusal traits. Finally, the present study has the inherent limitations of a cross-sectional design and can only determine the association between factors and malocclusion, but not their causal relationship. Therefore, future longitudinal studies are needed to dynamically observe the development of malocclusion and analyze the relationship between factors and malocclusion. Nevertheless, this study contributes to the existing literature, and epidemiologists and clinicians can gain a better understanding of the prevalence of malocclusion among preschool children in Huizhou. These findings will help in improving oral healthcare planning for deciduous-dentition malocclusions in the future.

## 5. Conclusions

The prevalence of malocclusion among 3- to 5-year-old children in Huizhou, China, was found to be high, with over half of the studied children affected. The prevalence of canine class III and anterior crossbite was shown to decrease with age. Deep overbite was the most prevalent malocclusion trait and was associated with dental caries in deciduous maxillary anterior teeth, as well as digit-sucking and lip-biting. Children with dental caries in anterior maxillary teeth had a lower prevalence of deep overbite. Children with a lip-biting habit had a higher prevalence of deep overbite compared to those without this deleterious oral habit, while children with a digit-sucking habit presented a lower prevalence. Additionally, dental caries were associated with spacing, lip-biting was related to increased overjet and crowding, and digit-sucking was associated with anterior open bite.

## Figures and Tables

**Table 1:** Prevalence of malocclusion in 3–5-year-old children in Huizhou, China.

Age and Sex	*n*	%	Malocclusion		*p* Value
			*n*	%	
Age					
3	170	11.7	133	78.2	0.001 *
4	743	51.1	518	69.7	
5	541	37.2	342	63.2	
Sex					
Boy	769	52.9	516	67.1	0.300
Girl	685	47.1	477	69.6	
Total	1454	100.0	993	68.3	

* *p* < 0.05.

**Table 2:** The composition and prevalence of occlusal traits.

Variable	Age 3 (Year)		Age 4 (Year)		Age 5 (Year)		Total		*p* Value
	*n*	%	*n*	%	*n*	%	*n*	%	
Molar relationship									
Flush terminal plane	96	56.5	442	59.5	310	57.3	848	58.3	0.524
Distal step	30	17.6	112	15.1	101	18.7	243	16.7	
Mesial step	44	25.9	189	25.4	130	24.0	363	25.0	
Canine relationship									
Class I	94	55.3 ^a^	483	65.0 ^a^	350	64.7 ^a^	927	63.8	0.008 * (b < a)
Class II	43	25.3 ^a^	164	22.1 ^a^	138	25.5 ^a^	345	23.7	
Class III	33	19.4 ^a^	96	12.9 ^a,b^	53	9.8 ^b^	182	12.5	
***Sagittal Anomalies***									
Increased overjet	18	10.6	92	12.4	72	13.3	182	12.5	0.638
Edge-to-edge incisor relationship	2	1.2	20	2.7	17	3.1	39	2.7	0.384
Anterior crossbite	22	12.9	66	8.9	25	4.6	113	7.8	0.001 *
***Vertical Anomalies***									
Deep overbite	92	54.1	368	49.5	247	45.7	707	48.6	0.122
Anterior open bite	2	1.2	10	1.3	5	0.9	17	1.2	0.786
***Transversal Anomalies***									
Posterior crossbite	1	0.6	1	0.1	0	0	2	0.1	-
Scissor bite	0	0	2	0.3	0	0	2	0.1	-
Midline deviation	14	8.2	56	7.5	47	8.7	117	8.0	0.752
***Space Discrepancies***									
Crowding	22	12.9	69	9.3	61	11.3	152	10.5	0.273
Spacing	80	47.1	398	53.6	293	54.2	771	53.0	0.247

* *p* < 0.05. a, b: Each superscript letter denotes a subset of age categories whose column proportions do not differ significantly from each other at the 0.05 level. b < a: The prevalence of class III canine relationships was significantly lower in the 5-year-old age group than that in the 3-year-old age group, and the prevalence of class III canine relationships was significantly lower than class I and II canine relationship among 5-year-old children.

**Table 3:** Occlusal traits and dental caries of preschool children in Huizhou, China.

Variable	Frequency (%)	Caries Prevalence	
		*n* (%)	*p* Value
Molar relationship			
Flush terminal plane	848 (58.3%)	619 (73.0%)	0.287
Distal step	243 (16.7%)	172 (70.8%)	
Mesial step	363 (25.0%)	277 (76.3%)	
Canine relationship			
Class I	927 (63.8%)	691 (74.5%)	0.058
Class II	345 (23.7%)	237 (68.7%)	
Class III	182 (12.5%)	140 (76.9%)	
***Sagittal Anomalies***			
Increased overjet			
Presence	182 (12.5%)	134 (73.6%)	0.955
Absence	1272 (87.5%)	934 (73.4%)	
Edge-to-edge incisor relationship			
Presence	39 (2.7%)	31 (79.5%)	0.387
Absence	1415 (97.3%)	1037 (73.3%)	
Anterior crossbite			
Presence	113 (7.8%)	79 (69.9%)	0.375
Absence	1341 (92.2%)	989 (73.8%)	
***Vertical Anomalies***			
Deep overbite			
Presence	707 (48.6%)	476 (67.3%)	<0.001 *
Absence	747 (51.4%)	592 (79.3%)	
Anterior open bite			
Presence	17 (1.2%)	12 (70.6%)	0.785
Absence	1437 (98.8%)	1056 (73.5%)	
***Transversal Anomalies***			
Posterior crossbite			
Presence	2 (0.1%)	1 (50.0%)	-
Absence	1452 (99.9%)	1067 (73.5%)	
Scissor bite			
Presence	2 (0.1%)	2 (100.0%)	-
Absence	1452 (99.9%)	1066 (73.4%)	
Midline deviation			
Presence	117 (8.0%)	89 (76.1%)	0.504
Absence	1337 (92.0%)	979 (73.2%)	
***Space Discrepancies***			
Crowding			
Presence	152 (10.5%)	115 (75.7%)	0.515
Absence	1302 (89.5%)	953 (73.2%)	
Spacing			
Presence	771 (53.0%)	532 (69.0%)	<0.001 *
Absence	683 (47.0%)	536 (78.5%)	

* *p* < 0.05.

**Table 4:** Association between deleterious oral habits and malocclusion.

Variable	Prevalence of Deep Overbite (Present/Total)	*p* Value	Prevalence of Increased Overjet (Present/Total)	*p* Value	Prevalence of Crowding (Present/Total)	*p* Value
Digit-sucking						
Presence	43.8% (179/409)	0.021 *	13.9% (57/409)	0.307	9.5% (39/409)	0.474
Absence	50.5% (528/1045)		12.0% (125/1045)		10.8% (113/1045)	
Lip-biting						
Presence	67.0% (75/112)	<0.001 *	20.5% (23/112)	0.009*	17.0% (19/112)	0.021 *
Absence	47.1% (632/1342)		11.8% (159/1342)		9.9% (133/1342)	
Tongue-thrusting						
Presence	48.6% (34/70)	0.993	10.0% (7/70)	0.515	10.0% (7/70)	0.899
Absence	48.6% (673/1384)		12.6% (175/1384)		10.5% (145/1384)	
Object-biting						
Presence	51.5% (102/198)	0.381	16.7% (33/198)	0.059	6.6% (13/198)	0.057
Absence	48.2% (605/1256)		11.9% (149/1256)		11.1% (139/1256)	
Bruxism						
Presence	46.6% (144/309)	0.423	15.5% (48/309)	0.072	10.0% (31/309)	0.785
Absence	49.2% (563/1145)		11.7% (134/1145)		10.6% (121/1145)	
Unilateral chewing						
Presence	49.2% (30/61)	0.929	6.6% (4/61)	0.160	8.2% (5/61)	0.557
Absence	48.6% (677/1393)		12.8% (178/1393)		10.6% (147/1393)	
Mouth-breathing						
Presence	44.2% (50/113)	0.333	13.3% (15/113)	0.800	13.3% (15/113)	0.309
Absence	49.0% (657/1341)		12.5% (167/1341)		10.2% (137/1341)	

* *p* < 0.05.

**Table 5:** Association between feeding/delivery methods and malocclusion.

Variable	Prevalence of Deep Overbite (Present/Total)	*p* Value	Prevalence of Increased Overjet (Present/Total)	*p* Value	Prevalence of Crowding (Present/Total)	*p* Value
Feeding methods in first 6 months						
exclusive breastfeeding	47.7% (353/740)	0.697	11.9% (88/740)	0.683	9.9% (73/740)	0.207
exclusive bottle-feeding	48.2% (92/191)		14.1% (27/191)		14.1% (27/191)	
mixed feeding	50.1% (262/523)		12.8% (67/523)		9.9% (52/523)	
Duration of breastfeeding						
Never	48.2% (92/191)	0.802	14.1% (27/191)	0.686	14.1% (27/191)	0.302
0–6 months	49.4% (196/397)		13.1% (52/397)		10.6% (42/397)	
6.1–12 months	49.5% (289/584)		12.5% (73/584)		9.2% (54/584)	
>12 months	46.1% (130/282)		10.6% (30/282)		10.3% (29/282)	
Duration of bottle-feeding						
0–12 months	50.5% (214/424)	0.602	12.3% (52/424)	0.703	10.1% (43/424)	0.935
12.1–18 months	48.8% (181/371)		13.7% (51/371)		10.2% (38/371)	
>18 months	47.3% (312/659)		12.0% (79/659)		10.8% (71/659)	
Use of pacifier						
Yes	46.3% (107/231)	0.445	13.0% (30/231)	0.814	12.6% (29/231)	0.256
No	49.1% (600/1223)		12.4% (152/1223)		10.1% (123/1223)	
Way of delivery						
Natural labor	48.8% (441/903)	0.835	12.0% (108/903)	0.411	11.0% (99/903)	0.417
Cesarean section	48.3% (266/551)		13.4% (74/551)		9.6% (53/551)	
Gestational age						
Preterm birth	54.1% (59/109)	0.151	17.4% (19/109)	0.247	10.1% (11/109)	0.772
Term birth	47.6% (595/1250)		12.2% (153/1250)		10.3% (129/1250)	
Posterm birth	55.8% (53/95)		10.5% (10/95)		12.6% (12/95)	

**Table 6:** Binary logistic regression analysis of relative factors and deep overbite.

Variable	Prevalence of Deep Overbite (Present/Total)	OR (95%CI)
Dental caries in anterior maxillary teeth		
Presence	58.5% (581/993)	0.70 (0.56–0.86)
Absence	66.2% (305/461)	
Digit-sucking		
Presence	43.8% (179/409)	0.73 (0.58–0.92)
Absence	50.5% (528/1045)	
Lip-biting		
Presence	67.0% (75/112)	2.36 (1.56–3.56)
Absence	47.1% (632/1342)	

## Data Availability

The datasets generated during and/or analyzed during the current study are available from the corresponding author upon reasonable request.

## Supplementary Materials

The following supporting information can be downloaded at: https://www.mdpi.com/article/10.3390/healthcare11071050/s1, Supplementary File S1: The Statement of Strengthening the Reporting of Observational Studies in Epidemiology (STROBE).

## References

[1] Lombardo G., Vena F., Negri P., Pagano S., Barilotti C., Paglia L., Colombo S., Orso M., Cianetti S. (2020). Worldwide prevalence of malocclusion in the different stages of dentition: A systematic review and meta-analysis. Eur. J. Paediatr. Dent..

[2] Amr-Rey O., Sanchez-Delgado P., Salvador-Palmer R., Cibrian R., Paredes-Gallardo V. (2022). Association between malocclusion and articulation of phonemes in early childhood. Angle Orthod..

[3] Santana L.G., Flores-Mir C., Iglesias-Linares A., Pithon M.M., Marques L.S. (2020). Influence of heritability on occlusal traits: A systematic review of studies in twins. Prog. Orthod..

[4] D’Onofrio L. (2019). Oral dysfunction as a cause of malocclusion. Orthod. Craniofac. Res..

[5] Bauman J.M., Souza J.G.S., Bauman C.D., Florio F.M. (2018). Epidemiological pattern of malocclusion in Brazilian preschoolers. Cienc. Saude Coletiva.

[6] Fu M., Zhang D., Wang B., Deng Y., Wang F., Ye X. (2002). The prevalence of malocclusion in China—An investigation of 25,392 children. Zhonghua Kou Qiang Yi Xue Za Zhi.

[7] Sepp H., Saag M., Vinkka-Puhakka H., Svedström-Oristo A.L., Peltomäki T. (2019). Occlusal traits of 4–5-year-old Estonians. Parents’ perception of orthodontic treatment need and satisfaction with dental appearance. Clin. Exp. Dent. Res..

[8] Berneburg M., Zeyher C., Merkle T., Möller M., Schaupp E., Göz G. (2010). Orthodontic findings in 4- to 6-year-old kindergarten children from southwest Germany. J. Orofac. Orthop..

[9] Dimberg L., Lennartsson B., Arnrup K., Bondemark L. (2015). Prevalence and change of malocclusions from primary to early permanent dentition: A longitudinal study. Angle Orthod..

[10] Antonini A., Marinelli A., Baroni G., Franchi L., Defraia E. (2005). Class II malocclusion with maxillary protrusion from the deciduous through the mixed dentition: A longitudinal study. Angle Orthod..

[11] Peres K.G., Peres M.A., Thomson W.M., Broadbent J., Hallal P.C., Menezes A.B. (2015). Deciduous-dentition malocclusion predicts orthodontic treatment needs later: Findings from a population-based birth cohort study. Am. J. Orthod. Dentofac. Orthop..

[12] (2020). Huizhou Municipal Bureau of Statistics. http://www.huizhou.gov.cn/hztjj/gkmlpt/content/4/4473/post_4473393.html#1030.

[13] Hakka Population. https://baijiahao.baidu.com/s?id=1709321196847038168&wfr=spider&for=pc.

[14] National Bureau of Statistics. http://www.gov.cn/xinwen/2022-01/17/content_5668815.htm.

[15] von Elm E., Altman D.G., Egger M., Pocock S.J., Gotzsche P.C., Vandenbroucke J.P., Initiative S. (2014). The Strengthening the Reporting of Observational Studies in Epidemiology (STROBE) Statement: Guidelines for reporting observational studies. Int. J. Surg..

[16] Serdar C.C., Cihan M., Yucel D., Serdar M.A. (2021). Sample size, power and effect size revisited: Simplified and practical approaches in pre-clinical, clinical and laboratory studies. Biochem. Med..

[17] Shen L., He F., Zhang C., Jiang H., Wang J. (2018). Prevalence of malocclusion in primary dentition in mainland China, 1988–2017: A systematic review and meta-analysis. Sci. Rep..

[18] Zhou X., Zhang Y., Wang Y., Zhang H., Chen L., Liu Y. (2017). Prevalence of Malocclusion in 3- to 5-Year-Old Children in Shanghai, China. Int. J. Environ. Res. Public Health.

[19] Zhou Z., Liu F., Shen S., Shang L., Shang L., Wang X. (2016). Prevalence of and factors affecting malocclusion in primary dentition among children in Xi’an, China. BMC Oral Health.

[20] Chen X., Xia B., Ge L. (2015). Effects of breast-feeding duration, bottle-feeding duration and non-nutritive sucking habits on the occlusal characteristics of primary dentition. BMC Pediatr..

[21] da Rosa D.P., Bonow M.L.M., Goettems M.L., Demarco F.F., Santos I.S., Matijasevich A., Barros A.J., Peres K.G. (2020). The influence of breastfeeding and pacifier use on the association between preterm birth and primary-dentition malocclusion: A population-based birth cohort study. Am. J. Orthod. Dentofac. Orthop..

[22] Foster T.D., Hamilton M.C. (1969). Occlusion in the primary dentition. Study of children at 2 and one-half to 3 years of age. Br. Dent. J..

[23] World Health Organization (2013). Oral Health Surveys Basic Methods.

[24] Ramos-Jorge J., Motta T., Marques L.S., Paiva S.M., Ramos-Jorge M.L. (2015). Association between anterior open bite and impact on quality of life of preschool children. Braz. Oral Res..

[25] Corrêa-Faria P., Ramos-Jorge M.L., Martins-Júnior P.A., Vieira-Andrade R.G., Marques L.S. (2014). Malocclusion in preschool children: Prevalence and determinant factors. Eur. Arch. Paediatr. Dent..

[26] Zhang S., Lo E.C.M., Chu C.H. (2017). Occlusal Features and Caries Experience of Hong Kong Chinese Preschool Children: A Cross-Sectional Study. Int. J. Environ. Res. Public Health.

[27] Peoples of Asia. https://www.britannica.com/topic/Hakka.

[28] Hakka Dialect. https://omniglot.com/chinese/hakka.htm.

[29] Hakka Cuisine. https://theculturetrip.com/asia/china/hong-kong/articles/an-introduction-to-traditional-hakka-cuisine/.

[30] Luo C., Duan L., Li Y., Xie Q., Wang L., Ru K., Nazir S., Jawad M., Zhao Y., Wang F. (2021). Insights From Y-STRs: Forensic Characteristics, Genetic Affinities, and Linguistic Classifications of Guangdong Hakka and She Groups. Front. Genet..

[31] Nakamura S., Miyajima K., Nagahara K., Killiany D.M., Tsuchiya T. (1999). Cephalometric changes during self-correction of primary anterior crossbite. ASDC J. Dent. Child..

[32] Srinivasan D., Loganathan D., Kumar S.S., Louis C.J., Eagappan S., Natarajan D. (2017). An Evaluation of Occlusal Relationship and Primate Space in Deciduous Dentition in Kancheepuram District, Tamil Nadu, India. J. Pharm. Bioallied Sci..

[33] Alkhtib A., Ghanim A., Temple-Smith M., Messer L.B., Pirotta M., Morgan M. (2016). Prevalence of early childhood caries and enamel defects in four and five-year old Qatari preschool children. BMC Oral Health.

[34] Cho V.Y., King N.M., Anthonappa R.P. (2021). Correlating spacing in the primary dentition and caries experience in preschool children. Eur. Arch. Paediatr. Dent..

[35] Subramaniam P., Babu Kl G., Nagarathna J. (2012). Interdental spacing and dental caries in the primary dentition of 4–6 year old children. J. Dent..

[36] Kasparaviciene K., Sidlauskas A., Zasciurinskiene E., Vasiliauskas A., Juodzbalys G., Sidlauskas M., Marmaite U. (2014). The prevalence of malocclusion and oral habits among 5–7-year-old children. Med. Sci. Monit..

[37] Chaiwat J., Deckunakorn S. (1991). Bite jumping appliance with lower lip bumper. J. Dent. Assoc. Thai.

[38] Fukumitsu K., Ohno F., Ohno T. (2003). Lip sucking and lip biting in the primary dentition: Two cases treated with a morphological approach combined with lip exercises and habituation. Int. J. Orofac. Myol..

[39] Rai A., Koirala B., Dali M., Shrestha S., Shrestha A., Niraula S.R. (2022). Prevalence of Oral Habits and its Association with Malocclusion in Primary Dentition among School Going Children of Nepal. J. Clin. Pediatr. Dent..

[40] Warren J.J., Bishara S.E. (2002). Duration of nutritive and nonnutritive sucking behaviors and their effects on the dental arches in the primary dentition. Am. J. Orthod. Dentofac. Orthop..

[41] Ling H.T.B., Sum F., Zhang L., Yeung C.P.W., Li K.Y., Wong H.M., Yang Y. (2018). The association between nutritive, non-nutritive sucking habits and primary dental occlusion. BMC Oral Health.

[42] Shah R., Ashley P., Amlani M., Noar J. (2021). Non-nutritive sucking habits in a child: A clinical protocol to their prevention and management. J. Orthod..

[43] Gianelly A.A. (2002). Treatment of crowding in the mixed dentition. Am. J. Orthod. Dentofac. Orthop..

[44] Foster T.D., Grundy M.C. (1986). Occlusal changes from primary to permanent dentitions. Br. J. Orthod..

[45] Bhat S.S., Rao H.A., Hegde K.S., Kumar B.K. (2012). Characteristics of primary dentition occlusion in preschool children: An epidemiological study. Int. J. Clin. Pediatr. Dent..

[46] Stahl F., Grabowski R., Gaebel M., Kundt G. (2007). Relationship between occlusal findings and orofacial myofunctional status in primary and mixed dentition. Part II: Prevalence of orofacial dysfunctions. J. Orofac. Orthop..

[47] da Silva Filho O.G., Santamaria M., Capelozza Filho L. (2007). Epidemiology of posterior crossbite in the primary dentition. J. Clin. Pediatr. Dent..

[48] Alhammadi M.S., Halboub E., Fayed M.S., Labib A., El-Saaidi C. (2018). Global distribution of malocclusion traits: A systematic review. Dent. Press J. Orthod..

[49] Stahl F., Grabowski R. (2004). Malocclusion and caries prevalence: Is there a connection in the primary and mixed dentitions?. Clin. Oral Investig..

[50] Al-Shahrani N., Al-Amri A., Hegazi F., Al-Rowis K., Al-Madani A., Hassan K.S. (2015). The prevalence of premature loss of primary teeth and its impact on malocclusion in the Eastern Province of Saudi Arabia. Acta Odontol. Scand..

